# Antiplane wave scattering from a cylindrical cavity in pre-stressed nonlinear elastic media

**DOI:** 10.1098/rspa.2015.0450

**Published:** 2015-10-08

**Authors:** Tom Shearer, William J. Parnell, I. David Abrahams

**Affiliations:** School of Mathematics, University of Manchester, Oxford Road, Manchester M13 9PL, UK

**Keywords:** pre-stress, nonlinear elasticity, scattering, inhomogeneous deformations, antiplane waves, elastomer

## Abstract

The effect of a longitudinal stretch and a pressure-induced inhomogeneous radial deformation on the scattering of antiplane elastic waves from a cylindrical cavity is determined. Three popular nonlinear strain energy functions are considered: the neo-Hookean, the Mooney–Rivlin and a two-term Arruda–Boyce model. A new method is developed to analyse and solve the governing wave equations. It exploits their properties to determine an asymptotic solution in the far-field, which is then used to derive a boundary condition to numerically evaluate the equations local to the cavity. This method could be applied to any linear ordinary differential equation whose inhomogeneous coefficients tend to a constant as its independent variable tends to infinity. The effect of the pre-stress is evaluated by considering the scattering cross section. A longitudinal stretch is found to decrease the scattered power emanating from the cavity, whereas a compression increases it. The effect of the pressure difference depends on the strain energy function employed. For a Mooney–Rivlin material, a cavity inflation increases the scattered power and a deflation decreases it; for a neo-Hookean material, the scattering cross section is unaffected by the radial deformation; and for a two-term Arruda–Boyce material, both inflation and deflation are found to decrease the scattered power.

## 1. Introduction

Canonical wave scattering problems have been of great interest for many decades, with applications in numerous areas, including water waves, electromagnetics, acoustics, seismology and non-destructive evaluation. The objective is to determine the scattered field given information regarding the form of the incident wave and scattering obstacle or *inhomogeneity*. In particular, the type of boundary conditions imposed on the boundary between the obstacle and the surrounding host medium is key [1,2]. The case of cavities, cracks and other defects in an elastic medium is one of tremendous importance [3–6] due to the fact that their presence frequently leads to a degradation in material performance. The primary aim of non-destructive evaluation is to predict the presence of such flaws in a medium and, although this is always framed as an inverse model, the forward scattering problem must be understood in order to deduce conclusions about the inverse problem [7]. In many cases there may well be a number of such inhomogeneities present in the medium, and so much effort has gone into the prediction of the so-called effective wavenumber of inhomogeneous media. This theory has been developed in the case of flawed media as well as fibre-reinforced or particulate composites, which themselves may possess imperfections associated with their adherence to the host phase (e.g. [8]).

The propagation of elastic waves in an inhomogeneous material depends strongly on the distribution and properties of the inhomogeneities inside the material. At low frequency, the medium generally responds as an effective homogeneous medium with properties defined by the leading order scattering characteristics of the inhomogeneities, i.e. the Rayleigh limit [9–11]. At higher frequencies, complex frequency-dependent behaviour can develop, for example, attenuation in random media [12] and complete reflection in periodic media in the so-called *band gaps* [13]. Inhomogeneous materials are implicitly required in order to construct *metamaterials*, media that are generally strongly anisotropic and frequency dependent [14,15]. Such media are generally not found in nature and enable rather surprising effects such as cloaking and negative refraction [16–18].

Recently, nonlinear elastic materials have been used in order to construct tunable band gaps that can be shifted in real time [19–22]. This work employs the theory of *small-on-large* [23,24] to derive the governing pressure-dependent incremental wave equations by linearizing about the nonlinear equilibrium state. Shearer *et al.* [25] used this theory to consider the propagation of torsional waves through an inhomogeneously pre-stressed annular cylinder. In the main, however, interest has centred on the influence of *homogeneous* stretch distributions (and hence the influence of induced anisotropy alone) on subsequent wave propagation (e.g. [26,27]). When the medium in question is *inhomogeneous* (for example, a fibre-reinforced, or particulate composite, material) and the host phase is nonlinear elastic, pre-stress will almost always lead to non-homogeneous as well as anisotropic stretch distributions, except in very special cases (e.g. [19]).

It is, therefore, of interest to investigate how an initial static pre-stress, which leads to inhomogeneous stress and strain fields in a nonlinear elastic body, influences the propagation and scattering of small-amplitude waves. In this article, we consider a canonical scattering problem, i.e. that of scattering of horizontally polarized shear waves from a cylindrical cavity in an incompressible, pre-stressed, nonlinear elastic medium. Note that a similar problem involving *homogeneous* initial deformations was studied by Leungvichcharoen & Wijeyewickrema [28]. We extend the work of Parnell & Abrahams [29], in which antiplane wave scattering from a cylindrical cavity in a neo-Hookean material was investigated, by considering more complex constitutive models, namely the Mooney–Rivlin and Arruda–Boyce strain energy functions. Parnell & Abrahams [29] showed that, in the neo-Hookean case, the scattering coefficients are completely unaffected by the application of a hydrostatic pressure in the radial direction along with a longitudinal stretch. Parnell and co-workers [30,31] exploited this result to create a theoretical *cloak* for antiplane elastic waves. The benefit of this over classical metamaterial cloaks is that inhomogeneous density is not required and the necessary inhomogeneity in the incremental shear modulus is induced naturally by the imposed pre-stress. Norris & Parnell [32] extended the work to the case of in-plane elastic waves, and Parnell & Shearer [15] considered the effect of an imperfect cloaking material characterized by a Mooney–Rivlin strain energy function. The governing equations could not be solved analytically in this latter case; they were solved numerically over the finite domain of the cloak. It is more challenging to solve the governing equations over an infinite domain, the case that shall be considered in this work. The developed methodology can be employed in general for any strain energy function. It also seems to be a plausible scheme for the general solution of ordinary differential equations with inhomogeneous coefficients.

Because scattering coefficients associated with single, canonical scattering problems are employed in multiple scattering models [8,11], it is envisaged that this work will be useful for the prediction of effective wave propagation through pre-stressed nonlinear elastic inhomogeneous media (e.g. soft porous materials), certainly for the case of dilute dispersions.

In §2, we describe the finite deformation that ensues within a nonlinear elastic material when a far-field hydrostatic pressure, along with a longitudinal stretch, is imposed. In §3, we then carry out the *small-on-large* analysis, assuming that the incremental field is a horizontally polarized (antiplane) shear wave and we derive the governing equation for the antiplane displacement field in the pre-stressed medium. In §4, we describe a novel hybrid analytical/numerical method used to solve the governing equations over an infinite domain and use it to determine, in §5, the scattering cross sections of the waves in neo-Hookean, Mooney–Rivlin and Arruda–Boyce materials subjected to various levels of pre-stress. Concluding remarks are offered in §6.

## 2. Initial finite static deformation (pre-stress)

Consider an isotropic, *incompressible*, nonlinear elastic material of infinite extent with a circular cylindrical cavity, of initial radius *A*, whose axis is parallel to the *Z* axis of a Cartesian coordinate system (*X*,*Y*,*Z*) with its centre in the (*X*,*Y*) plane located at the origin. The constitutive behaviour of the hyperelastic material is described by the strain energy function *W*=*W*(*I*_1_,*I*_2_) [23], where *I*_*j*_, *j*=1,2,3 are the principal strain invariants, and we note that *W* is independent of *I*_3_ due to the constraint of incompressibility (i.e. *I*_3_=1 for all deformations).

**Figure 1.**
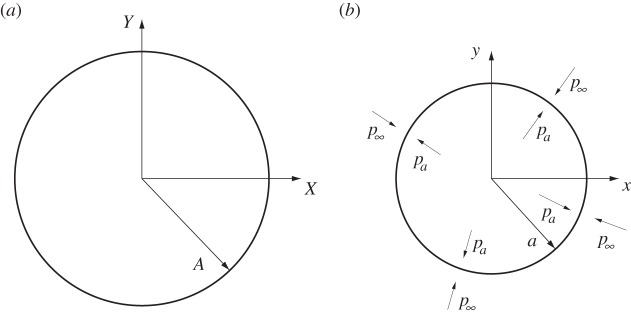
(*a*) The undeformed cavity, with radius *A*. (*b*) The deformed cavity, with radius *a*. The deformation is due to an imposed far-field hydrostatic pressure $p_{\infty }$ and an internal pressure *p*_*a*_, along with an axial stretch *ζ*.

The medium is deformed due to an imposed far-field hydrostatic pressure, an internal hydrostatic pressure and a uniform axial stretch, as depicted in figure 1. The symmetry of geometry and forcing therefore implies that the deformation is described by

2.1 $$R=R(r),\;\Theta =\theta \;\mathrm{and}\;Z=\frac{z}{\zeta },$$

where (*R*,*Θ*,*Z*) and (*r*,*θ*,*z*) are cylindrical polar coordinates (X=Rcos⁡Θ, Y=Rsin⁡Θ, x=rcos⁡θ, y=rsin⁡θ) in the undeformed and deformed configurations, respectively, and *R*(*r*) is a function that is determined from the radial equation of equilibrium and incompressibility condition. Note the convention introduced in (2.1), i.e. that upper case variables correspond to the undeformed configuration, whereas lower case variables correspond to the deformed configuration. We are interested in incremental perturbations from the initial statically deformed state and, therefore, it will be convenient to derive equations in terms of coordinates in the deformed configuration. Position vectors in the undeformed and deformed configurations are, respectively,

2.2 $$\text{X}=R{\text{E}}_{R}(\Theta )+Z{\text{E}}_{Z}=R(r){\text{E}}_{R}(\Theta )+\frac{z}{\zeta }{\text{E}}_{Z},\;\text{x}=r{\text{e}}_{r}(\theta )+z{\text{e}}_{z},$$

where **E**_*R*_ and **E**_*Z*_ are the radial and longitudinal basis vectors in the undeformed configuration, and **e**_*r*_ and **e**_*z*_ are the corresponding basis vectors in the deformed configuration.

Using (2.1), it can be shown that the principal stretches for this deformation in the radial, azimuthal and longitudinal directions, respectively, are

2.3 $$\lambda_{r}=\frac{dr}{dR}=\frac{1}{R^{\prime }(r)},\;\lambda_{\theta }=\frac{r}{R(r)}\;\mathrm{and}\;\lambda_{z}=\zeta .$$

The deformation gradient tensor **F** is given by

2.4 F=Grad x,

where Grad represents the gradient operator in the undeformed configuration. In our case, we have

2.5 $$\text{F}=F_{iJ}{\text{e}}_{i}⊗{\text{E}}_{J},\;F_{iJ}=\left(\begin{array}{ccc} \lambda_{r}(r) & 0 & 0\\ 0 & \lambda_{\theta }(r) & 0\\ 0 & 0 & \lambda_{z} \end{array}\right)=\left(\begin{array}{ccc} {\left(R^{\prime }(r)\right)}^{-1} & 0 & 0\\ 0 & \frac{r}{R(r)} & 0\\ 0 & 0 & \zeta \end{array}\right).$$

For an incompressible material $J=\sqrt{I_{3}}=\det\text{F}=1$ and so

2.6 $$\lambda_{r}\lambda_{\theta }\lambda_{z}=\frac{\zeta r}{R(r)R^{\prime }(r)}=1.$$

This is easily solved to yield

2.7 $$R(r)=\sqrt{\zeta (r^{2}+M)},$$

where *M* is a constant defined by

2.8 $$M=\frac{A^{2}}{\zeta }-a^{2}.$$

From Ogden [23,24], the Cauchy and nominal stress tensors for an incompressible material are, respectively, given, in terms of the deformation gradient and strain energy function, by

2.9 $$\text{T}=\text{F}\frac{\partial W}{\partial \text{F}}+Q\text{I}\;\mathrm{and}\;\text{S}=\frac{\partial W}{\partial \text{F}}+Q{\text{F}}^{-1},$$

where **I** is the identity tensor, *Q* is a Lagrange multiplier associated with the incompressibility constraint, often referred to as an *arbitrary hydrostatic pressure*, and we note that the differentiation of *W* with respect to **F** is defined component-wise as follows:

2.10 $${\left(\frac{\partial W}{\partial \text{F}}\right)}_{Ij}=\frac{\partial W}{\partial F_{jI}}.$$

The static equations of equilibrium are then given by

2.11 div T=0,

where div signifies the divergence operator with respect to **x**. These reduce to

2.12 $$\frac{\partial T_{rr}}{\partial r}+\frac{1}{r}(T_{rr}-T_{\theta \theta })=0,\;\frac{\partial T_{\theta \theta }}{\partial \theta }=0\;\mathrm{and}\;\frac{\partial T_{zz}}{\partial z}=0,$$

where **T**=*T*_*ij*_**e**_*i*_⊗**e**_*j*_. The second and third of these imply *Q*=*Q*(*r*). Integrating the first of (2.12), using (2.9) and applying *T*_*rr*_|_*r*=*a*_=−*p*_*a*_, we find that the radial stress is given by

2.13 $$T_{rr}(r)+p_{a}=\int_{a}^{r}\frac{1}{s}\left(\lambda_{\theta }(s)\frac{\partial W}{\partial \lambda_{\theta }}-\lambda_{r}(s)\frac{\partial W}{\partial \lambda_{r}}\right)ds,$$

then by applying $T_{rr}\to -p_{\infty }$ as r→∞, we obtain an equation relating the pressure difference to the resulting deformation,

2.14 $$p_{a}-p_{\infty }=\int_{a}^{\infty }\frac{1}{s}\left(\lambda_{\theta }(s)\frac{\partial W}{\partial \lambda_{\theta }}-\lambda_{r}(s)\frac{\partial W}{\partial \lambda_{r}}\right)ds.$$

To proceed, we must select a strain energy function *W* to substitute into equation (2.14). We shall use three different strain energy functions, the neo-Hookean:

2.15 $$W_{\mathrm{NH}}=\frac{\mu }{2}(I_{1}-3)=\frac{\mu }{2}(\lambda_{r}^{2}+\lambda_{\theta }^{2}+\lambda_{z}^{2}-3),$$

where *μ* is the ground-state shear modulus of the material under consideration, the Mooney–Rivlin:

2.16 $$\begin{array}{rl} W_{\mathrm{MR}} & =\frac{\mu }{2}(C_{1}(I_{1}-3)+C_{2}(I_{2}-3))\\ & =\frac{\mu }{2}(C_{1}(\lambda_{r}^{2}+\lambda_{\theta }^{2}+\lambda_{z}^{2}-3)+C_{2}(\lambda_{r}^{2}\lambda_{\theta }^{2}+\lambda_{r}^{2}\lambda_{z}^{2}+\lambda_{\theta }^{2}\lambda_{z}^{2}-3)), \end{array}$$

where *C*_1_ and *C*_2_ are material constants that sum to unity, and the Arruda–Boyce model. The Arruda–Boyce strain energy function can be expressed as an infinite series in terms of powers of *I*_1_ [33]. For simplicity, we shall use the first two terms only:

2.17 $$\begin{array}{rl} W_{\mathrm{AB}} & =\frac{5N\mu }{3+5N}\left(\frac{1}{2}(I_{1}-3)+\frac{1}{20N}(I_{1}^{2}-9)\right)\\ & =\frac{5N\mu }{3+5N}\left(\frac{1}{2}(\lambda_{r}^{2}+\lambda_{\theta }^{2}+\lambda_{z}^{2}-3)+\frac{1}{20N}(\lambda_{r}^{4}+\lambda_{\theta }^{4}+\lambda_{z}^{4}+2(\lambda_{r}^{2}\lambda_{\theta }^{2}+\lambda_{r}^{2}\lambda_{z}^{2}+\lambda_{\theta }^{2}\lambda_{z}^{2})-9)\right), \end{array}$$

where *N* is the assumed number of links in the polymer chains that form the molecular basis of rubber-like materials and we note that the coefficient in front of the shear modulus is necessary for this two-term model to be consistent with linear elasticity.

The resulting pressure–deformation relationship in the neo-Hookean case is

2.18 $$\frac{p_{\infty }-p_{a}}{\mu }=\frac{1}{2\zeta }\left(\log\left(\frac{A^{2}}{\zeta a^{2}}\right)+\frac{A^{2}}{\zeta a^{2}}-1\right),$$

in the Mooney–Rivlin case is

2.19 $$\frac{p_{\infty }-p_{a}}{\mu }=\frac{C_{1}+\zeta^{2}C_{2}}{2\zeta }\left(\log\left(\frac{A^{2}}{\zeta a^{2}}\right)+\frac{A^{2}}{\zeta a^{2}}-1\right),$$

and in the Arruda–Boyce case is

2.20 $$\begin{array}{rl} \frac{p_{\infty }-p_{a}}{\mu } & =\frac{1}{4(3+5N)\zeta^{4}}\left(1-\frac{\zeta a^{2}}{A^{2}}\right)\left(\frac{A^{4}}{a^{4}}+2\zeta^{2}+\frac{A^{2}}{a^{2}}\zeta (3+10N\zeta +2\zeta^{3})\right)\\ & \;+\frac{1+5N\zeta +\zeta^{3}}{2(3+5N)}\log\left(\frac{A^{2}}{\zeta a^{2}}\right). \end{array}$$

**Figure 2.**
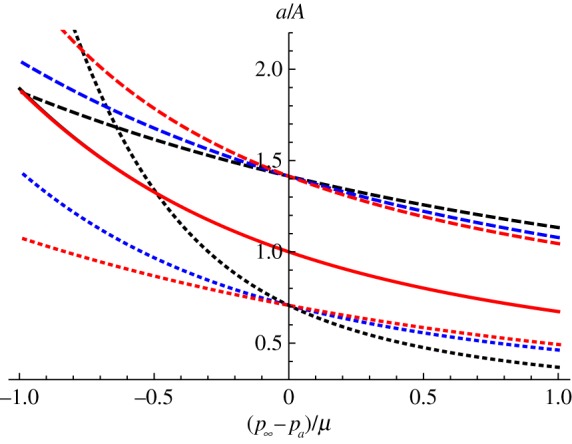
*a*/*A* as a function of $(p_{\infty }-p_{a})/\mu$. The black curves represent the neo-Hookean strain energy function, the blue curves represent the Mooney–Rivlin strain energy function and the red curves represent the Arruda–Boyce strain energy function. The solid lines correspond to *ζ*=1, the dashed to $\zeta =\frac{1}{2}$ and the dotted to *ζ*=2. The values chosen for the constants in the Mooney–Rivlin strain energy function were *C*_1_=0.724, *C*_2_=0.276, and the value chosen for *N* in the Arruda–Boyce strain energy function was *N*=26.5.

In figure 2, we plot *a*/*A* as a function of $(p_{\infty }-p_{a})/\mu$ for each of the strain energy functions listed above, and for three separate values of *ζ*. The black curves represent the neo-Hookean strain energy function, the blue curves represent the Mooney–Rivlin strain energy function and the red curves represent the Arruda–Boyce strain energy function. The solid lines correspond to *ζ*=1, the dashed to $\zeta =\frac{1}{2}$ and the dotted to *ζ*=2. The values chosen for the constants in the Mooney–Rivlin strain energy function were *C*_1_=0.724, *C*_2_=0.276, which were the values used by Mooney [34] to fit Gerke's [35] experimental data on the elongation of vulcanized rubber. The value chosen for *N* in the Arruda–Boyce strain energy function was *N*=26.5. This value of *N* was used by Arruda & Boyce [33] to match Treloar's [36] data on the uniaxial extension, biaxial extension and shear of vulcanized rubber. Note that, for *ζ*=1, the neo-Hookean and Mooney–Rivlin curves coincide and the Arruda–Boyce curve is so close to them that it also appears to lie on top of them in the figure.

## 3. Incremental deformations

We now examine incremental deformations from the deformed state *b*. To this end, consider a finite deformation of the original body *B* to a new deformed state $\bar{b}$ which is close to the configuration *b*. The position vector in the new deformed state $\bar{b}$ is given by $\bar{\text{x}}$ and we define

3.1 $$\text{u}=\bar{\text{x}}-\text{x},\;|\text{u}|\ll 1$$

as the difference between position vectors in $\bar{b}$ and *b*. Since $\bar{b}$ is close to *b*, **u** is called an *incremental displacement* which we assume to be time dependent. For the purposes of this article we assume that the incremental deformation is of *antiplane* type and time-harmonic, i.e.

3.2 $$\text{u}=ℜ[w(r,\theta )\exp(-i\omega t)]{\text{e}}_{z},$$

where the notation ℜ indicates that the real part of the expression inside the square brackets is to be taken. We next use the theory of *small-on-large* (see appendix A), to determine the governing equation of motion,

3.3 $$\frac{1}{r}\frac{\partial }{\partial r}\left(r\mu_{r}(r)\frac{\partial w}{\partial r}\right)+\frac{\mu_{\theta }(r)}{r^{2}}\frac{\partial^{2}w}{\partial \theta^{2}}+\rho \omega^{2}w=0,$$

where

3.4 $$\begin{array}{rl} \mu_{r}(r) & =\left(\frac{\lambda_{r}(\partial W/\partial \lambda_{r})-\lambda_{z}(\partial W/\partial \lambda_{z})}{\lambda_{r}^{2}-\lambda_{z}^{2}}\right)\lambda_{r}^{2}\\ \text{and}\;\;\mu_{\theta }(r) & =\left(\frac{\lambda_{\theta }(\partial W/\partial \lambda_{\theta })-\lambda_{z}(\partial W/\partial \lambda_{z})}{\lambda_{\theta }^{2}-\lambda_{z}^{2}}\right)\lambda_{\theta }^{2}. \end{array}\}$$

Given an incident field that takes plane wave form in the far-field, i.e.

3.5 $$w^{i}∼e^{ikx}=\sum_{n=0}^{\infty }\varepsilon_{n}i^{n}J_{n}(kr)\cos(n\theta )$$

as r→∞, where *J*_*n*_ is the Bessel function of the first kind of order *n* and

3.6 $$\varepsilon_{n}=\left\{ \begin{array}{ll} 1 & n=0,\\ 2 & n\neq 0, \end{array}\right.$$

we seek solutions to (3.3) in the form

3.7 $$w(r,\theta )=\sum_{n=0}^{\infty }\varepsilon_{n}i^{n}f_{n}(r)\cos(n\theta ).$$

As such for the *n*th mode, we have

3.8 $$\frac{1}{r}\frac{\partial }{\partial r}(r\mu_{r}(r)f_{n}^{\prime }(r))+\left(\rho \omega^{2}-\frac{\mu_{\theta }(r)}{r^{2}}n^{2}\right)f_{n}(r)=0,$$

in which *prime* notation denotes differentiation with respect to *r*. Clearly different incident fields could be considered as desired. By substituting (2.15)–(2.17) into (3.4), we can obtain explicit expressions for the anisotropic shear moduli *μ*_*r*_(*r*) and *μ*_*θ*_(*r*), which can then be used to determine the antiplane wave governing equation for each strain energy function. In the neo-Hookean case, we have

3.9 $$\left(1+\frac{M}{r^{2}}\right)f_{n}^{\prime \prime }(r)+\frac{1}{r}\left(1-\frac{M}{r^{2}}\right)f_{n}^{\prime }(r)+\left(k_{\mathrm{NH}}^{2}-\frac{n^{2}}{r^{2}+M}\right)f_{n}(r)=0,$$

where

3.10 $$k_{\mathrm{NH}}^{2}=\zeta K^{2}\;\mathrm{and}\;K^{2}=\frac{\rho \omega^{2}}{\mu },$$

in the Mooney–Rivlin case we have

3.11 $$\left(1+\frac{m}{r^{2}}\right)f_{n}^{\prime \prime }(r)+\frac{1}{r}\left(1-\frac{m}{r^{2}}\right)f_{n}^{\prime }(r)+\left(k_{\mathrm{MR}}^{2}-\frac{n^{2}}{r^{2}}\left(1-\frac{m}{r^{2}+M}\right)\right)f_{n}(r)=0,$$

where

3.12 $$k_{\mathrm{MR}}^{2}=\frac{\zeta^{2}K^{2}}{T},\;m=\frac{M\zeta C_{1}}{T},\;T=1+(\zeta -1)C_{1},$$

and in the Arruda–Boyce case we have

3.13 $$\begin{array}{rl} & \left(1+\frac{M}{r^{2}}+\chi \frac{M^{2}}{r^{4}}\right)f_{n}^{\prime \prime }(r)+\frac{1}{r}\left(1-\frac{M}{r^{2}}-3\chi \frac{M^{2}}{r^{4}}\right)f_{n}^{\prime }(r)\\ & \;+\left(k_{\mathrm{AB}}^{2}-n^{2}\left(\frac{1}{r^{2}+M}+\frac{\chi M^{2}}{r^{2}{\left(r^{2}+M\right)}^{2}}\right)\right)f_{n}(r)=0, \end{array}$$

where

3.14 $$k_{\mathrm{AB}}^{2}=(3+5N)\zeta^{2}\chi K^{2}\;\mathrm{and}\;\chi =\frac{1}{2+5N\zeta +\zeta^{3}},$$

and we note that, when *ζ*=1, *k*_NH_=*k*_MR_=*k*_AB_=*K*.

By substituting (3.7) into (A 20), we see that the boundary condition on *r*=*a* reduces to

3.15 $$f_{n}^{\prime }(a)=0,$$

for all *n*. This boundary condition remains the same regardless of the strain energy function used to characterize the material under consideration.

We note that the effect of the radial pre-stress on the governing wave equations (3.9)–(3.13) decreases as r→∞, and in the limit as *M*/*r*^2^→0, (3.9), (3.11) and (3.13) become

3.16 $$f_{n}^{\prime \prime }(r)+\frac{1}{r}f_{n}^{\prime }(r)+\left(k^{2}-\frac{n^{2}}{r^{2}}\right)f_{n}(r)=0,$$

where *k*^2^=*k*^2^_NH_ in the neo-Hookean case, *k*^2^=*k*^2^_MR_ in the Mooney–Rivlin case, and *k*^2^=*k*^2^_AB_ in the Arruda–Boyce case. Equation (3.16) is the standard governing equation for antiplane waves in a stress-free elastic medium (albeit with a modified wavenumber) and is readily solved:

3.17 $$f_{n}(r)=f_{n}^{i}(r)+f_{n}^{s}(r),\;f_{n}^{i}(r)=J_{n}(kr)\;\mathrm{and}\;f_{n}^{s}(r)=a_{n}H_{n}^{\left(1\right)}(kr),$$

where we identify $f_{n}^{i}(r)$ as the *n*th component of the incoming plane wave and thus equate it to *J*_*n*_ via (3.5). Furthermore, $f_{n}^{s}(r)$ is the *n*th component of the scattered wave, represented by a Hankel function of the first kind, $H_{n}^{\left(1\right)}(kr)$, and *a*_*n*_ is the scattering coefficient associated with this *n*th outgoing wave term.

Owing to the complexity of the above incremental governing equations, they cannot be solved via standard methods, except for a neo-Hookean material (see §3a). In this case, exact solutions can be found [29]; however, in the Mooney–Rivlin (except when *n*=0, see §3b) and Arruda–Boyce cases, we must instead find an approximate solution. The method used to do this is described in §4.

### (a) The special case of neo-Hookean media

Parnell & Abrahams [29] showed that (3.9) can be solved analytically to give

3.18 $$f_{n}(r)=J_{n}\left(K\sqrt{\zeta (r^{2}+M)}\right)+a_{n}H_{n}^{\left(1\right)}\left(K\sqrt{\zeta (r^{2}+M)}\right)$$

for the case of an incoming plane wave, where the scattering coefficients *a*_*n*_ are given by

3.19 $$a_{n}=-\frac{J_{n}^{\prime }\left(K\sqrt{\zeta (a^{2}+M)}\right)}{H_{n}^{(1)\prime }\left(K\sqrt{\zeta (a^{2}+M)}\right)}=-\frac{J_{n}^{\prime }(KA)}{H_{n}^{(1)\prime }(KA)}.$$

This is the form the scattering coefficients take in a stress-free medium containing a cavity of radius *A*, i.e. the radius of the *undeformed* cavity in the problem being considered in this paper. Therefore, in the neo-Hookean case, rather surprisingly, all the scattering coefficients are completely unaffected by the pre-stress.

### (b) Analytical solution in the Mooney–Rivlin case when *n*=0

In the Mooney–Rivlin case, to the authors’ knowledge, a general solution for all mode numbers *n* cannot be found; however, we note that, when *n*=0, (3.11) reduces to

3.20 $$\left(1+\frac{m}{r^{2}}\right)f_{0}^{\prime \prime }(r)+\frac{1}{r}\left(1-\frac{m}{r^{2}}\right)f_{0}^{\prime }(r)+k^{2}f_{0}(r)=0,$$

which *can* be solved analytically (as for the neo-Hookean case) to give

3.21 $$f_{0}(r)=c_{1}J_{0}\left(k\sqrt{r^{2}+m}\right)+c_{2}H_{0}^{\left(1\right)}\left(k\sqrt{r^{2}+m}\right),$$

where *c*_1_ and *c*_2_ are arbitrary constants. Upon matching this solution to the far-field form (3.17), we obtain *c*_1_=1 and *c*_2_=*a*_0_, and applying the boundary condition (3.15), we obtain

3.22 $$a_{0}=-\frac{J_{0}^{\prime }\left(k\sqrt{a^{2}+m}\right)}{H_{0}^{(1)\prime }\left(k\sqrt{a^{2}+m}\right)}.$$

This is a similar form to that which the neo-Hookean scattering coefficients take when *n*=0; however, since $k\sqrt{a^{2}+m}\neq KA$, the above expression *is* dependent on the pre-stress. To determine the effect of the pre-stress on the other modes (*n*≠0), however, we must use the method described in the following section.

## 4. Solution method

As mentioned above, the Mooney–Rivlin (3.11) and Arruda–Boyce (3.13) governing equations are not readily solved via standard methods. Hence, to proceed, we recall that, in the limit as *M*/*r*^2^→0, an analytical solution (3.17) to the governing equations may be found. Therefore, for large *r*, we should be able to derive an approximate solution. For a given pre-stress parameter *M*, we shall choose a large radius *b* such that *M*/*b*^2^=*ϵ*^2^≪1 and derive an approximate solution to the governing equation for *r*≥*b*. Then, in the region *r*∈[*a*,*b*], we shall solve the governing equations numerically, with the boundary conditions on *r*=*b* being dictated by enforcing continuity of displacement and traction with the approximate solution. In what follows we describe a ‘general’ recipe for numerically evaluating the solution in the far-field to *O*(*ϵ*^2^), which will work for any strain energy function; for ease of exposition this is presented for a Mooney–Rivlin material, but the Arruda–Boyce case follows in a very similar fashion. Note, however, in the Mooney–Rivlin case we can in fact evaluate the integral expressions below analytically, as will be discussed at the end of this section. We begin by introducing new independent and dependent variables *s*=*r*/*b* and *F*_*n*_(*s*)=*f*_*n*_(*r*), respectively, so that (3.11) can be rewritten as

4.1 $$\left(1+\frac{m}{M}\frac{\epsilon^{2}}{s^{2}}\right)\frac{d^{2}F_{n}}{ds^{2}}+\frac{1}{s}\left(1-\frac{m}{M}\frac{\epsilon^{2}}{s^{2}}\right)\frac{dF_{n}}{ds}+\left(\kappa^{2}-\frac{n^{2}}{s^{2}}\left(1-\frac{(m/M)\epsilon^{2}}{s^{2}+\epsilon^{2}}\right)\right)F_{n}=0,$$

where *κ*^2^=(*kb*)^2^. Given (3.17), we then assume the following (regular) expansion for *F*_*n*_(*s*):

4.2 $$F_{n}(s)=J_{n}(\kappa s)+a_{n}H_{n}^{\left(1\right)}(\kappa s)+\epsilon^{2}G_{n}(s)+O(\epsilon^{4}),$$

so that at *O*(1) we obtain

4.3 $$\begin{array}{rl} & \frac{d^{2}}{ds^{2}}(J_{n}(\kappa s))+\frac{1}{s}\frac{d}{ds}(J_{n}(\kappa s))+\left(\kappa^{2}-\frac{n^{2}}{s^{2}}\right)J_{n}(\kappa s)\\ & \;+a_{n}\left(\frac{d^{2}}{ds^{2}}\left(H_{n}^{\left(1\right)}(\kappa s)\right)+\frac{1}{s}\frac{d}{ds}\left(H_{n}^{\left(1\right)}(\kappa s)\right)+\left(\kappa^{2}-\frac{n^{2}}{s^{2}}\right)H_{n}^{\left(1\right)}(\kappa s)\right)=0, \end{array}$$

which is satisfied trivially, and at *O*(*ϵ*^2^) we obtain

4.4 $$\begin{array}{rl} & \frac{d^{2}G_{n}}{ds^{2}}+\frac{1}{s}\frac{dG_{n}}{ds}+\left(\kappa^{2}-\frac{n^{2}}{s^{2}}\right)G_{n}\\ & \;=\frac{m}{M}\left(\frac{2}{s^{3}}\frac{d}{ds}\left(J_{n}(\kappa s)+a_{n}H_{n}^{\left(1\right)}(\kappa s)\right)+\left(\frac{\kappa^{2}}{s^{2}}-\frac{2n^{2}}{s^{4}}\right)\left(J_{n}(\kappa s)+a_{n}H_{n}^{\left(1\right)}(\kappa s)\right)\right). \end{array}$$

Now, if we let

4.5 $$G_{n}(s)=\frac{m}{M}H_{n}^{\left(1\right)}(\kappa s)g_{n}(s),$$

then (4.4) simplifies to

4.6 $$\begin{array}{rl} \frac{d}{ds}\left(s{\left(H_{n}^{\left(1\right)}(\kappa s)\right)}^{2}\frac{dg_{n}}{ds}\right) & =\frac{2}{s^{2}}H_{n}^{\left(1\right)}(\kappa s)\frac{d}{ds}(J_{n}(\kappa s))+\left(\frac{\kappa^{2}}{s}-\frac{2n^{2}}{s^{3}}\right)H_{n}^{\left(1\right)}(\kappa s)J_{n}(\kappa s)\\ & \;+a_{n}\left(\frac{2}{s^{2}}H_{n}^{\left(1\right)}(\kappa s)\frac{d}{ds}(H_{n}^{\left(1\right)}(\kappa s))+\left(\frac{\kappa^{2}}{s}-\frac{2n^{2}}{s^{3}}\right){\left(H_{n}^{\left(1\right)}(\kappa s)\right)}^{2}\right). \end{array}$$

In the limit as s→∞, the governing equation (4.1) reduces to Bessel's equation (since the terms multiplying *m*/*M* disappear), whose solution is $F_{n}(s)=J_{n}(\kappa s)+a_{n}H_{n}^{\left(1\right)}(\kappa s)$; hence *G*_*n*_ and *g*_*n*_ must tend to 0 as s→∞. The next step of the method, therefore, is to integrate (4.6) from *s* to ∞, and divide by $s{\left(H_{n}^{\left(1\right)}(\kappa s)\right)}^{2}$, to obtain

4.7 $$\begin{array}{rl} \frac{dg_{n}}{ds} & =-\frac{1}{s{\left(H_{n}^{\left(1\right)}(\kappa s)\right)}^{2}}\int_{s}^{\infty }\left(\frac{2}{x^{2}}H_{n}^{\left(1\right)}(\kappa x)\frac{d}{dx}(J_{n}(\kappa x))+\left(\frac{\kappa^{2}}{x}-\frac{2n^{2}}{x^{3}}\right)H_{n}^{\left(1\right)}(\kappa x)J_{n}(\kappa x)\right)dx\\ & \;+\frac{a_{n}}{s^{3}}-\frac{a_{n}}{s{\left(H_{n}^{\left(1\right)}(\kappa s)\right)}^{2}}\int_{s}^{\infty }\left(\frac{\kappa^{2}}{x}+\frac{2(1-n^{2})}{x^{3}}\right){\left(H_{n}^{\left(1\right)}(\kappa x)\right)}^{2}\;dx, \end{array}$$

and, upon integrating again, we find

4.8 $$\begin{array}{rl} g_{n}(s) & =\int_{s}^{\infty }\frac{1}{y{\left(H_{n}^{\left(1\right)}(\kappa y)\right)}^{2}}\int_{y}^{\infty }\left(\frac{2}{x^{2}}H_{n}^{\left(1\right)}(\kappa x)\frac{d}{dx}(J_{n}(\kappa x))+\left(\frac{\kappa^{2}}{x}-2\frac{n^{2}}{x^{3}}\right)H_{n}^{\left(1\right)}(\kappa x)J_{n}(\kappa x)\right)dx\;dy-\frac{a_{n}}{2s^{2}}\\ & \;+a_{n}\int_{s}^{\infty }\frac{1}{y{\left(H_{n}^{\left(1\right)}(\kappa y)\right)}^{2}}\int_{y}^{\infty }\left(\frac{\kappa^{2}}{x}+\frac{2(1-n^{2})}{x^{3}}\right){\left(H_{n}^{\left(1\right)}(\kappa x)\right)}^{2}\;dx\;dy. \end{array}$$

So,

4.9 $$F_{n}(s)=J_{n}(\kappa s)+a_{n}H_{n}^{\left(1\right)}(\kappa s)+\frac{m}{M}\epsilon^{2}H_{n}^{\left(1\right)}(\kappa s)g_{n}(s)+O(\epsilon^{4}),$$

and, therefore,

4.10 $$f_{n}(r)=J_{n}(kr)+a_{n}H_{n}^{\left(1\right)}(kr)+\frac{m}{M}\epsilon^{2}H_{n}^{\left(1\right)}(kr)g_{n}\left(\frac{r}{b}\right)+O(\epsilon^{4}),$$

and, hence,

4.11 $$f_{n}(b)=J_{n}(kb)+a_{n}H_{n}^{\left(1\right)}(kb)+\frac{m}{b^{2}}H_{n}^{\left(1\right)}(kb)g_{n}(1)+O(\epsilon^{4})$$

and

4.12 $$\begin{array}{rl} {\frac{df_{n}}{dr}|}_{r=b} & ={\frac{d}{dr}(J_{n}(kr))|}_{r=b}+a_{n}{\frac{d}{dr}(H_{n}^{\left(1\right)}(kr))|}_{r=b}+\frac{m}{b^{3}}H_{n}^{\left(1\right)}(kb){\frac{dg_{n}}{ds}|}_{s=1}\\ & \;+\frac{m}{b^{2}}{\frac{d}{dr}(H_{n}^{\left(1\right)}(kr))|}_{r=b}g_{n}(1)+O(\epsilon^{4}). \end{array}$$

Now, in order to determine the appropriate boundary condition on *r*=*b*, we require a suitable choice for *a*_*n*_. We note that we can write

4.13 $$f_{n}(b)=\alpha a_{n}+\beta ,$$

where

4.14 $$\begin{array}{rl} \alpha & =H_{n}^{\left(1\right)}(kb)\left(1-\frac{m}{2b^{2}}+\frac{m}{b^{2}}\int_{1}^{\infty }\frac{1}{y{\left(H_{n}^{\left(1\right)}(\kappa y)\right)}^{2}}\int_{y}^{\infty }\left(\frac{\kappa^{2}}{x}+\frac{2(1-n^{2})}{x^{3}}\right){\left(H_{n}^{\left(1\right)}(\kappa x)\right)}^{2}\;dx\;dy\right), \end{array}$$

4.15 $$\begin{array}{rl} \beta & =J_{n}(kb)+\frac{m}{b^{2}}H_{n}^{\left(1\right)}(kb)\int_{1}^{\infty }\frac{1}{y{\left(H_{n}^{\left(1\right)}(\kappa y)\right)}^{2}}\\ & \;\times \int_{y}^{\infty }\left(\frac{2}{x^{2}}H_{n}^{\left(1\right)}(\kappa x)\frac{d}{dx}(J_{n}(\kappa x))+\left(\frac{\kappa^{2}}{x}-\frac{2n^{2}}{x^{3}}\right)H_{n}^{\left(1\right)}(\kappa x)J_{n}(\kappa x)\right)dx\;dy \end{array}$$

4.16 $$\begin{array}{rl} \text{and}\;f_{n}^{\prime }(b) & ={\frac{df_{n}}{dr}|}_{r=b}=\gamma a_{n}+\delta , \end{array}$$

where

4.17 $$\begin{array}{rl} \gamma & ={\frac{d}{dr}(H_{n}^{\left(1\right)}(kr))|}_{r=b}\frac{\alpha }{H_{n}^{\left(1\right)}(kb)}+\frac{m}{b^{3}}H_{n}^{\left(1\right)}(kb)\\ & \;\times \left(1-\frac{1}{{\left(H_{n}^{\left(1\right)}(kb)\right)}^{2}}\int_{1}^{\infty }\left(\frac{\kappa^{2}}{x}+\frac{2(1-n^{2})}{x^{3}}\right){\left(H_{n}^{\left(1\right)}(\kappa x)\right)}^{2}\;dx\right) \end{array}$$

and

4.18 $$\begin{array}{rl} \delta & ={\frac{d}{dr}(J_{n}(kr))|}_{r=b}-{\frac{d}{dr}(H_{n}^{\left(1\right)}(kr))|}_{r=b}\frac{J_{n}(kb)}{H_{n}^{\left(1\right)}(kb)}+{\frac{d}{dr}(H_{n}^{\left(1\right)}(kr))|}_{r=b}\frac{\beta }{H_{n}^{\left(1\right)}(kb)}\\ & \;-\frac{m}{b^{3}H_{n}^{\left(1\right)}(kb)}\int_{1}^{\infty }\left(\frac{2}{x^{2}}H_{n}^{\left(1\right)}(\kappa x)\frac{d}{dx}(J_{n}(\kappa x))+\left(\frac{\kappa^{2}}{x}-\frac{2n^{2}}{x^{3}}\right)H_{n}^{\left(1\right)}(\kappa x)J_{n}(\kappa x)\right)dx. \end{array}$$

Solving (4.13) and (4.14) for *a*_*n*_, we obtain

4.19 $$a_{n}=\frac{1}{\Delta }(\delta f_{n}(b)-\beta f_{n}^{\prime }(b)),$$

where

4.20 Δ=αδ−βγ,

and eliminating *a*_*n*_ from (4.13) and (4.16), we obtain our final boundary condition,

4.21 $$\gamma f_{n}(b)-\alpha f_{n}^{\prime }(b)=-\Delta .$$

The method is now clear. We choose *b* such that *M*/*b*^2^≪1, and numerically solve equation (3.11) subject to (3.15) and (4.21). Once solved, we use the values of *f*_*n*_(*b*) and $f_{n}^{\prime }(b)$ determined by our numerical solver in equation (4.19) to deduce the value of *a*_*n*_. The predicted value of *a*_*n*_ will depend on the chosen value of *b*, therefore we must increase *b* until the solution has converged to a desired level of accuracy.

We note that, owing to their highly oscillatory nature, the integrals in equations (4.14)–(4.18) are difficult to evaluate numerically; however, in appendix B, we show how they can be rearranged into forms that are more readily evaluated. A similar procedure can be followed to obtain such integrals for other strain energy functions. In fact, for Mooney-Rivlin materials it transpires that the integrals can be evaluated exactly, leading to an explicit expression for the *O*(*ϵ*^2^) term:

4.22 $$G_{n}(s)=\frac{m}{M}\frac{1}{2s}\frac{d}{ds}(J_{n}(\kappa s)+a_{n}H_{n}^{\left(1\right)}(\kappa s)).$$

This has allowed us to verify that the numerical procedure is effective.

**Figure 3.**
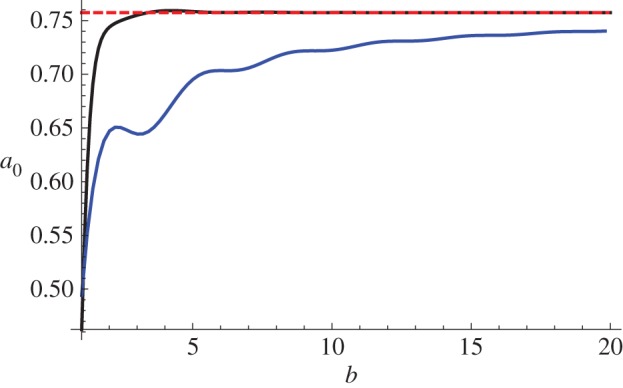
The fundamental scattering coefficient *a*_0_ in a pre-stressed neo-Hookean material with *M*=1, *ζ*=1, *K*=1 and *a*=1, as a function of *b*, derived using the method described in §4 (black), the analytic expression (red, dashed) and a simpler version of the method in which only the *O*(1) terms in the far-field expansion are included (blue).

To demonstrate how the method described above converges with increasing *b*, in figure 3 we plot the fundamental scattering coefficient *a*_0_ in a pre-stressed neo-Hookean material with *M*=1, *ζ*=1, *K*=1 and *a*=1. The black line gives the values derived using the method above (in which we determined the far-field solution up to *O*(*ϵ*^2^)), the red dashed line shows the analytical solution (which is available since we are considering a neo-Hookean material), and the blue line represents the solution that is obtained when one neglects the *O*(*ϵ*^2^) correction to *f*_*n*_(*r*) (see (4.2)) in the far-field. We observe that the inclusion of the *O*(*ϵ*^2^) terms greatly improves the convergence. We discuss the convergence of the method further in appendix C.

## 5. Scattering cross sections

In order to determine the effect of the pre-stress on the scattered waves, we plot the non-dimensionalized scattering cross section (see appendix D),

5.1 $$q=\frac{2}{kA}\sum_{n=0}^{\infty }\varepsilon_{n}|a_{n}{|}^{2}.$$

This quantity gives the ratio of the time-averaged power emitted from a scatterer to the intensity of the incoming wave, scaled on the diameter of the scatterer. In the absence of a scatterer, this must obviously be equal to zero.

### (a) The effect of the applied pressure difference

**Figure 4.**
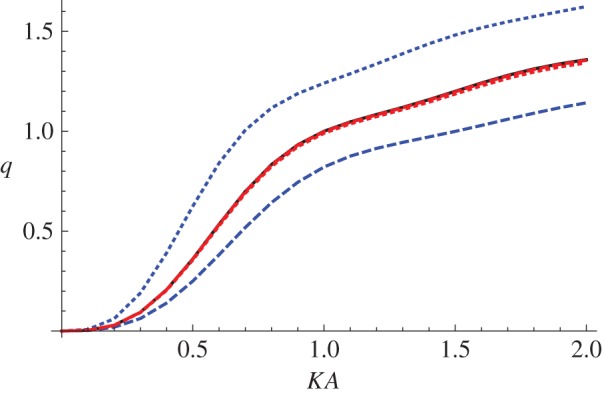
The scattering cross section of a neo-Hookean (black), a Mooney–Rivlin with *C*_1_=0.724, *C*_2_=0.276 (blue) and an Arruda–Boyce material with *N*=26.5 (red) for three values of the non-dimensionalized pressure difference: $(p_{\infty }-p_{a})/\mu =0$ (solid), $(p_{\infty }-p_{a})/\mu =1$ (dashed) and $(p_{\infty }-p_{a})/\mu =-1$ (dotted) as a function of *KA*. The value of the longitudinal stretch was chosen to be *ζ*=1 and the initial cavity radius was taken to be *A*=1.

In figure 4, we plot the scattering cross section of a neo-Hookean material (black), a Mooney–Rivlin material with *C*_1_=0.724, *C*_2_=0.276 (blue) and an Arruda–Boyce material with *N*=26.5 (red), for three values of the non-dimensionalized pressure difference: $(p_{\infty }-p_{a})/\mu =0$ (solid), $(p_{\infty }-p_{a})/\mu =1$ (dashed) and $(p_{\infty }-p_{a})/\mu =-1$ (dotted), as a function of *KA*. The values of the deformed radii *a* that result from these pre-stress values are shown in table 1. The value of the longitudinal stretch was chosen to be *ζ*=1 and the initial cavity radius was taken to be *A*=1. The numerical solver used to evaluate equations (3.11) and (3.13) was NDSolve in Mathematica 9.0 (Wolfram Research, Inc.) and the value selected for the outer radius of the numerical domain was *b*=80 in all cases.

**Table 1.** The effect of the applied pressure differences on the deformed radius *a* for the three strain energy functions, given a longitudinal stretch *ζ*=1 and undeformed radius *A*=1.

strain energy function	applied pressure difference	deformed radius *a*
neo-Hookean	$(p_{\infty }-p_{a})/\mu =0$	1
neo-Hookean	$(p_{\infty }-p_{a})/\mu =1$	0.672987
neo-Hookean	$(p_{\infty }-p_{a})/\mu =-1$	1.89503
Mooney–Rivlin	$(p_{\infty }-p_{a})/\mu =0$	1
Mooney–Rivlin	$(p_{\infty }-p_{a})/\mu =1$	0.672987
Mooney–Rivlin	$(p_{\infty }-p_{a})/\mu =-1$	1.89503
Arruda–Boyce	$(p_{\infty }-p_{a})/\mu =0$	1
Arruda–Boyce	$(p_{\infty }-p_{a})/\mu =1$	0.67336
Arruda–Boyce	$(p_{\infty }-p_{a})/\mu =-1$	1.88936

**Figure 5.**
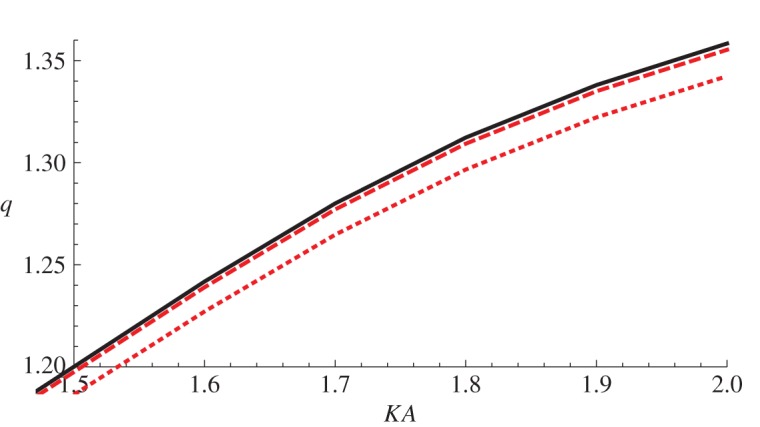
An enlarged version of the region 1.5≤*KA*≤2, 1.2≤*q*≤1.36 displayed in figure 4.

We note that, as discussed earlier, all the neo-Hookean scattering cross sections and the stress-free scattering cross sections of the other materials coincide. In the Mooney–Rivlin material, a greater pressure at infinity than on *r*=*a* leads to a decrease in scattered energy, whereas a negative pressure difference increases the scattering. This is intuitive since a positive pressure difference decreases the size of the scatterer and a negative pressure difference increases it. Interestingly, however, in the Arruda–Boyce material *any non-zero pressure difference* (positive or negative) causes a small *decrease* in scattering (counterintuitively, the effect is more pronounced for a negative pressure difference). This can be seen in figure 5, which shows an enlarged version of the region 1.5≤*KA*≤2, 1.2≤*q*≤1.36 in figure 4. As the Arruda–Boyce strain energy function is essentially a higher order correction to a neo-Hookean strain energy function, involving the invariant *I*_1_ only, it is perhaps not surprising that the deviation of the behaviour of this material from neo-Hookean is small; however, the reversal of the effect of a negative pressure difference as compared with a Mooney–Rivlin material is somewhat surprising.

### (b) The effect of the applied longitudinal stretch

**Figure 6.**
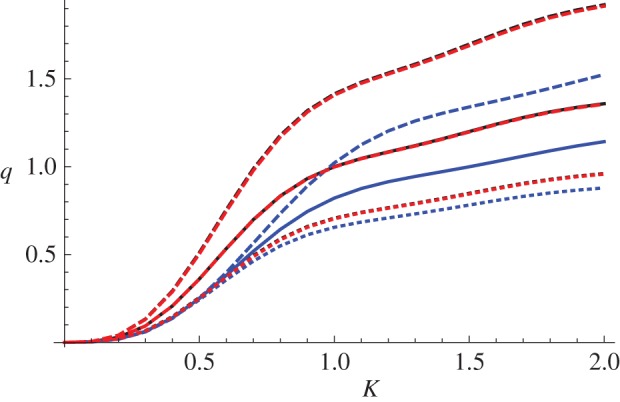
The scattering cross section of a neo-Hookean (black), a Mooney–Rivlin with *C*_1_=0.724, *C*_2_=0.276 (blue) and an Arruda–Boyce material with *N*=26.5 (red) for three values of the longitudinal stretch: *ζ*=1 (solid), $\zeta =\frac{1}{2}$ (dashed) and *ζ*=2 (dotted), as a function of *KA*. The value of the non-dimensionalized pressure difference was chosen to be $(p_{\infty }-p_{a})/\mu =1$ and the initial cavity radius was chosen to be *A*=1.

In figure 6, we plot the scattering cross section of a neo-Hookean material (black), a Mooney–Rivlin material with *C*_1_=0.724, *C*_2_=0.276 (blue) and an Arruda–Boyce material with *N*=26.5 (red), for three values of the longitudinal stretch: *ζ*=1 (solid), $\zeta =\frac{1}{2}$ (dashed) and *ζ*=2 (dotted), as a function of *KA*. The values of the deformed radii *a* that result from these pre-stress values are tabulated in table 2. The value of the non-dimensionalized pressure difference was chosen to be $(p_{\infty }-p_{a})/\mu =1$ and the initial cavity radius was chosen to be *A*=1. Once again, the numerical solver used to solve equations (3.11) and (3.13) was NDSolve in Mathematica 9.0 (Wolfram Research, Inc.) and the value selected for the outer radius of the numerical domain was *b*=80.

**Table 2.** The effect of the applied longitudinal stretches on the deformed radius *a* for each strain energy function used, given a non-dimensionalized pressure difference $(p_{\infty }-p_{a})/\mu =1$ and undeformed radius *A*=1.

strain energy function	applied longitudinal stretch	deformed radius *a*
neo-Hookean	*ζ*=1	0.672987
neo-Hookean	$\zeta =\frac{1}{2}$	1.13331
neo-Hookean	*ζ*=2	0.367933
Mooney–Rivlin	*ζ*=1	0.672987
Mooney–Rivlin	$\zeta =\frac{1}{2}$	1.07858
Mooney–Rivlin	*ζ*=2	0.462386
Arruda–Boyce	*ζ*=1	0.67336
Arruda–Boyce	$\zeta =\frac{1}{2}$	1.04534
Arruda–Boyce	*ζ*=2	0.492763

In all cases, a longitudinal stretch leads to a decrease in the scattered power, whereas a longitudinal compression increases it. This effect could have been predicted by observing that the scattering cross section is scaled on the deformed wavenumber *k*, which increases with increasing stretch and decreases with increasing compression (see equations (3.10), (3.12) and (3.14)). Once again, we observe that the Arruda–Boyce scattering cross sections take values very close to those found for the neo-Hookean strain energy function.

## 6. Discussion

In this paper, we have investigated the effect of pre-stress on the scattering of antiplane elastic waves from a cylindrical cavity. The governing equations have inhomogeneous coefficients, and, as a result, could not be solved analytically except for special cases. In order to analyse their behaviour, therefore, a new hybrid analytical–numerical method was developed which relies upon the fact that the inhomogeneities in the coefficients approach zero as r→∞, thus allowing an asymptotic solution to be derived for large *r*. The asymptotic solution was used to determine a boundary condition for a numerical solver in the region around the cavity. This hybrid method could be applied to any linear ordinary differential equation whose inhomogeneous coefficients tend to a constant as its independent variable tends to infinity.

In order to analyse the effect of the pre-stress, the scattering cross section was plotted for several values of the longitudinal stretch and pressure difference. It was shown that a positive longitudinal stretch led to a decrease in scattered power, whereas a compression caused an increase. The effect of the pressure difference, however, was more interesting. It was shown that, for a Mooney–Rivlin material, a positive pressure difference led to a decrease in scattered power, whereas a negative pressure difference led to an increase. This is intuitive as one would expect that inflating a cavity would increase its ability to scatter waves, whereas a deflation should decrease that ability. In the neo-Hookean case, however, the scattered power was completely unaffected by the pre-stress, whereas, in the Arruda–Boyce case, any non-zero pressure difference (positive or negative) led to a decrease in scattering. This result was not expected; however, it can be explained by considering the form of the strain energy function that was used. The two-term Arruda–Boyce model introduces an *O*(*M*^2^) modification to the neo-Hookean incremental equation (this can be seen by comparing equation (3.13) with equation (3.9)). Since *M*^2^ is positive for any real *M*, these new terms are insensitive to whether *M* is positive (corresponding to a cavity compression) or negative (corresponding to a cavity inflation) and therefore always reduce the scattering. It is possible that, by expanding the Arruda–Boyce model to third order in *I*_1_, the introduction of *O*(*M*^3^) terms in the governing incremental equation would reverse this behaviour; however, it is likely that these terms would be so small (due to the *O*(1/*N*^2^) coefficients that multiply the *O*(*M*^3^) terms—see equation (2.17) for reference) that the *O*(*M*^2^) terms would still dominate. In either case, the difference between the Arruda–Boyce and the neo-Hookean material response is expected to be small.

We note that the neo-Hookean and Arruda–Boyce strain energy functions are independent of the second strain invariant *I*_2_, and can therefore be classified as ‘*I*_1_ models’. It is well known that *I*_1_ models display unphysical behaviour in many circumstances. For example, they do not exhibit the Poynting effect [37]. It appears that the problem raised here for the Arruda–Boyce model is another example of the deficiencies of such strain energy functions.

## Data accessibility

This work does not have any experimental data. Calculations were performed using Mathematica v. 9.0.

## Appendix A. Summary of small-on-large theory

For the small-on-large details, we follow the approach described in Ogden [23] and Destrade & Saccomandi [38], with slight modifications to notation. We consider incremental deformations from the deformed body *b*. To this end, consider a finite deformation of the original body *B* to a new deformed state $\bar{b}$ which is close to the configuration *b*. We shall call this new configuration the *perturbed configuration*. Position vectors in the deformed states *b* and $\bar{b}$ are defined by **x** and $\bar{\text{x}}$, respectively, and we define

A 1 $$\text{u}=\bar{\text{x}}-\text{x},\;|\text{u}|\ll 1$$

as the difference between position vectors in $\bar{b}$ and *b*. Since $\bar{b}$ is close to *b*, **u** is called an *incremental displacement* which, in our case, is time dependent. We assume that the time-harmonic incremental deformation is an *antiplane wave*, i.e.

A 2 $$\text{u}=w(x,y)\;e^{-i\omega t}{\text{e}}_{z},$$

where **e**_*z*_ is a unit vector in the *z*-direction in the body *b*.

Given the deformation gradient (2.4), we can define the additional deformation gradients $\text{f}=\mathrm{grad}\bar{\text{x}}$ and $\bar{\text{F}}=\mathrm{Grad}\bar{\text{x}}=\text{f}\text{F}$, where grad represents the gradient operator with respect to the *statically* deformed configuration. Therefore,

A 3 $$\Gamma =\mathrm{Grad}\;\text{u}=\mathrm{Grad}(\bar{\text{x}}-\text{x})=(\text{f}-\text{I})\text{F}$$

and similarly *γ*=grad **u**=**f**−**I**, so that **f**=**I**+*γ* and *Γ*=*γ***F**.

Furthermore, given the stresses (2.9) in the configuration *b*, we can define the corresponding stresses $\bar{\text{T}}=\text{T}+\tau$ and $\bar{\text{S}}=\text{S}+s$ in $\bar{b}$, where ***τ*** and **s** are the incremental Cauchy and nominal stresses, respectively. By taking an expansion about the deformation **F**, it is straightforward to show that for an incompressible material

A 4 $$\text{s}=\text{L}:\gamma \text{F}+q{\text{F}}^{-1}-Q{\text{F}}^{-1}\gamma ,$$

where *q* is the increment of *Q* and **L**=∂^2^*W*/∂**F**^2^ defined componentwise via *L*_*IjKl*_=∂^2^*W*/∂*F*_*jI*_∂*F*_*lK*_. The colon notation indicates the double contraction **A**:**b**=*A*_*ijkl*_*b*_*lk*_. Defining the *push-forward of the incremental nominal stress* as *ζ*=**F****s**, from (A 4) this therefore has the form

A 5 ζ=M:γ+qI−Qγ,

where the components of the instantaneous modulus tensor **M** are defined by

A 6 $$M_{ijkl}=\frac{\partial^{2}W}{\partial F_{jM}\partial F_{lN}}F_{iM}F_{kN}.$$

Convenient forms for this tensor have been given by Ogden [23] as

A 7 $$\begin{array}{rlr} 2M_{iijj} & =\lambda_{i}\lambda_{j}W_{ij}=M_{jjii}, & \;\text{no sum on }i,j, \end{array}$$

A 8 $$\begin{array}{rlr} M_{ijij} & =\left(\frac{\lambda_{i}W_{i}-\lambda_{j}W_{j}}{\lambda_{i}^{2}-\lambda_{j}^{2}}\right)\lambda_{i}^{2}, & \;i\neq j,\lambda_{i}\neq \lambda_{j}, \end{array}$$

A 9 $$\begin{array}{rlr} M_{ijij} & =\frac{1}{2}(M_{iiii}-M_{iijj}+\lambda_{i}W_{i}), & \;i\neq j,\;\lambda_{i}=\lambda_{j} \end{array}$$

A 10 $$\begin{array}{rlr} \text{and}\;M_{ijji} & =M_{jiij}=M_{ijij}-\lambda_{i}W_{i}, & \;i\neq j, \end{array}$$

where *W*_*i*_=∂*W*/∂λ_*i*_ and *W*_*ij*_=∂^2^*W*/∂λ_*i*_∂λ_*j*_. Analysis of the Cauchy stress gives rise to the relationship

A 11 τ=ζ+γT,

for an incompressible material.

The equations of motion in body $\bar{b}$ in terms of the Cauchy stress are

A 12 $$\bar{\mathrm{div}}\;\bar{\text{T}}=\bar{\rho }\frac{\partial^{2}\bar{\text{U}}}{\partial t^{2}}=\rho \frac{\partial^{2}\text{u}}{\partial t^{2}},$$

where $\bar{\mathrm{div}}$ represents the divergence operator in the perturbed configuration. The second of the above equalities holds as the body is incompressible (i.e. $\bar{\rho }=\rho$) and because $\bar{\text{U}}=\text{U}+\text{u}$ where **U** is not time dependent. Using the fact that div **T**=**0**, it is relatively straightforward to show that

A 13 $$\bar{\mathrm{div}}\;\bar{\text{T}}=\mathrm{div}\;(\tau -\gamma \text{T})=\mathrm{div}\;\zeta ,$$

where we have used the relationship between ***ζ*** and ***τ*** above. The incremental equations are therefore

A 14 $$\mathrm{div}\;\zeta =\rho \frac{\partial^{2}\text{u}}{\partial t^{2}}.$$

If we substitute (A 2) into the above, two of the equations imply that *q* is independent of *r* and *θ*, and the single remaining governing equation of motion is

A 15 $$\frac{1}{r}\frac{\partial }{\partial r}(r\zeta_{rz})+\frac{1}{r}\frac{\partial \zeta_{\theta z}}{\partial \theta }+\rho \omega^{2}w+\frac{\partial q}{\partial z}=0,$$

where

A 16 $$\zeta_{rz}=M_{rzrz}\gamma_{zr}\;\mathrm{and}\;\zeta_{\theta z}=M_{\theta z\theta z}\gamma_{z\theta }.$$

In this case *τ*_*rz*_=*ζ*_*rz*_ and *τ*_*θz*_=*ζ*_*θz*_ and so the instantaneous moduli *M*_*rzrz*_ and *M*_*θzθz*_ can be interpreted as pressure-dependent anisotropic shear moduli, given by

A 17 $$M_{rzrz}(r)=\mu_{r}(r)=\left(\frac{\lambda_{r}W_{r}-\lambda_{z}W_{z}}{\lambda_{r}^{2}-\lambda_{z}^{2}}\right)\lambda_{r}^{2}\;\mathrm{and}\;M_{\theta z\theta z}(r)=\mu_{\theta }(r)=\left(\frac{\lambda_{\theta }W_{\theta }-\lambda_{z}W_{z}}{\lambda_{\theta }^{2}-\lambda_{z}^{2}}\right)\lambda_{\theta }^{2}.$$

Using (A 16) with (A 17) in (A 15) gives the governing equation of motion in terms of the displacement *w*, which is

A 18 $$\frac{1}{r}\frac{\partial }{\partial r}\left(r\mu_{r}(r)\frac{\partial w}{\partial r}\right)+\frac{\mu_{\theta }(r)}{r^{2}}\frac{\partial^{2}w}{\partial \theta^{2}}+\rho \omega^{2}w+\frac{\partial q}{\partial z}=0.$$

We now apply the condition that the pressure on the surface of the cavity (i.e. where *r*=*a*) remains unaltered by the perturbation, so that

A 19 $$\bar{\text{T}}\bar{\text{n}}=-p\bar{\text{n}},$$

where $\bar{\text{n}}$ is the outer unit normal in the perturbed configuration. This condition reduces to

A 20 $${\frac{\partial w}{\partial r}|}_{r=a}=0,\;q=0,$$

and the second of these equations allows us to reduce (A 18) to

A 21 $$\frac{1}{r}\frac{\partial }{\partial r}\left(r\mu_{r}(r)\frac{\partial w}{\partial r}\right)+\frac{\mu_{\theta }(r)}{r^{2}}\frac{\partial^{2}w}{\partial \theta^{2}}+\rho \omega^{2}w=0.$$

## Appendix B. Integrals

Here we describe how to numerically evaluate *α*, *β*, *γ* and *δ* defined in equations (4.14)–(4.18). We begin by noting that *γ* has two integrals of the form

B 1 $$I_{p}=\int_{1}^{\infty }\frac{{\left(H_{n}^{\left(1\right)}(\kappa x)\right)}^{2}}{x^{p}}\;dx,\;p=1,3.$$

The integrand here is highly oscillatory and so is not easy to evaluate numerically. We note, however, that the singularities of $H_{n}^{\left(1\right)}(\kappa x)$ lie entirely in the lower half of the complex *x*-plane. Let us write *u*=*κx*, so that

B 2 $$I_{p}=\kappa^{p-1}\int_{\kappa }^{\infty }\frac{{\left(H_{n}^{\left(1\right)}(u)\right)}^{2}}{u^{p}}\;du.$$

Then, we note that, in the limit as |u|→∞,

B 3 $${\left(H_{n}^{\left(1\right)}(u)\right)}^{2}∼-\frac{2i}{\pi u}{\left(-1\right)}^{n}\;e^{2iu},$$

therefore we can rotate the contour in *I*_*p*_ into the upper half-plane:

B 4 $$I_{p}=\kappa^{p-1}\int_{\kappa }^{i\infty }\frac{{\left(H_{n}^{\left(1\right)}(u)\right)}^{2}}{u^{p}}\;du.$$

This form can now be easily evaluated numerically. Similarly, we note that *α* has integrals of the form

B 5 $$L_{p}=\int_{1}^{\infty }\frac{1}{y{\left(H_{n}^{\left(1\right)}(\kappa y)\right)}^{2}}\int_{y}^{\infty }\frac{{\left(H_{n}^{\left(1\right)}(\kappa x)\right)}^{2}}{x^{p}}\;dx\;dy,\;p=1,3.$$

Upon making the substitutions *u*=*κx*, *v*=*κy* and again rotating into the upper half-plane, we obtain

B 6 $$\begin{array}{rl} L_{p} & =\kappa^{p-1}\int_{\kappa }^{\infty }\frac{1}{v{\left(H_{n}^{\left(1\right)}(v)\right)}^{2}}\int_{v}^{\infty }\frac{{\left(H_{n}^{\left(1\right)}(u)\right)}^{2}}{u^{p}}\;du\;dv\\ & =\kappa^{p-1}\int_{\kappa }^{\infty }\frac{1}{v{\left(H_{n}^{\left(1\right)}(v)\right)}^{2}}\int_{v}^{i\infty }\frac{{\left(H_{n}^{\left(1\right)}(u)\right)}^{2}}{u^{p}}\;du\;dv, \end{array}$$

which, again, is readily computable. Furthermore, *δ* contains integrals that can be written as

B 7 $$\begin{array}{rl} J_{p} & =\int_{1}^{\infty }\frac{H_{n}(\kappa x)J_{n}(\kappa x)}{x^{p}}\;dx=\kappa^{p-1}\int_{\kappa }^{\infty }\frac{H_{n}^{\left(1\right)}(u)J_{n}(u)}{u^{p}}\;du\\ & =\frac{\kappa^{p-1}}{2}\int_{\kappa }^{\infty }\frac{{\left(H_{n}^{\left(1\right)}(u)\right)}^{2}+H_{n}^{\left(1\right)}(u)H_{n}^{\left(2\right)}(u)}{u^{p}}\;du\\ & =\frac{I_{p}}{2}+\frac{\kappa^{p-1}}{2}\int_{\kappa }^{\infty }\frac{H_{n}^{\left(1\right)}(u)H_{n}^{\left(2\right)}(u)}{u^{p}}\;du,\;p=1,3, \end{array}$$

where $H_{n}^{\left(2\right)}(u)$ is the Hankel function of the second kind. The last of these integrals is not oscillatory, and so can be numerically evaluated as is. The other integral in *δ* can be written as

B 8 $$\begin{array}{rl} J & =\int_{1}^{\infty }\frac{1}{x^{2}}H_{n}^{\left(1\right)}(\kappa x)\frac{d}{dx}(J_{n}(\kappa x))\;dx=\frac{\kappa }{2}\int_{1}^{\infty }\frac{1}{x^{2}}H_{n}^{\left(1\right)}(\kappa x)(J_{n-1}(\kappa x)-J_{n+1}(\kappa x))\;dx\\ & =\frac{\kappa^{2}}{4}\int_{\kappa }^{i\infty }\frac{1}{u^{2}}H_{n}^{\left(1\right)}(u)(H_{n-1}^{\left(1\right)}(u)-H_{n+1}^{\left(1\right)}(u))\;du+\frac{\kappa^{2}}{4}\int_{\kappa }^{\infty }\frac{1}{u^{2}}H_{n}^{\left(1\right)}(u)(H_{n-1}^{\left(2\right)}(u)-H_{n+1}^{\left(2\right)}(u))\;du. \end{array}$$

Finally, the double integrals in *β* can be written as

B 9 $$\begin{array}{rl} M_{p} & =\int_{1}^{\infty }\frac{1}{y{\left(H_{n}^{\left(1\right)}(\kappa y)\right)}^{2}}\int_{y}^{\infty }\frac{H_{n}^{\left(1\right)}(\kappa x)J_{n}(\kappa x)}{x^{p}}\;dx\;dy\\ & =\kappa^{p-1}\int_{\kappa }^{\infty }\frac{1}{v{\left(H_{n}^{\left(1\right)}(v)\right)}^{2}}\int_{v}^{\infty }\frac{H_{n}^{\left(1\right)}(u)J_{n}(u)}{u^{p}}\;du\;dv\\ & =\frac{\kappa^{p-1}}{2}\int_{\kappa }^{\infty }\frac{1}{v{\left(H_{n}^{\left(1\right)}(v)\right)}^{2}}\int_{v}^{i\infty }\frac{{\left(H_{n}^{\left(1\right)}(u)\right)}^{2}}{u^{p}}\;du\;dv\\ & \;+\frac{\kappa^{p-1}}{2}\int_{\kappa }^{\infty }\frac{1}{v{\left(H_{n}\left(1\right)(v)\right)}^{2}}\int_{v}^{\infty }\frac{H_{n}^{\left(1\right)}(u)H_{n}^{\left(2\right)}(u)}{u^{p}}\;du\;dv\\ & =\frac{L_{p}}{2}+\frac{\kappa^{p-1}}{2}\int_{\kappa }^{\infty }\frac{1}{v{\left(H_{n}\left(1\right)(v)\right)}^{2}}\int_{v}^{\infty }\frac{H_{n}^{\left(1\right)}(u)H_{n}^{\left(2\right)}(u)}{u^{p}}\;du\;dv,\;p=1,3, \end{array}$$

and

B 10 $$\begin{array}{rl} P & =\int_{1}^{\infty }\frac{1}{y{\left(H_{n}^{\left(1\right)}(\kappa y)\right)}^{2}}\int_{y}^{\infty }\frac{1}{x^{2}}H_{n}^{\left(1\right)}(\kappa x)\frac{d}{dx}(J_{n}(\kappa x))\;dx\;dy\\ & =\kappa^{2}\int_{\kappa }^{\infty }\frac{1}{v{\left(H_{n}^{\left(1\right)}(v)\right)}^{2}}\int_{v}^{\infty }\frac{1}{u^{2}}H_{n}^{\left(1\right)}(u)\frac{d}{du}(J_{n}(u))\;du\;dv\\ & =\frac{\kappa^{2}}{4}\int_{\kappa }^{\infty }\frac{1}{v{\left(H_{n}^{\left(1\right)}(v)\right)}^{2}}(\int_{v}^{i\infty }\frac{H_{n}^{\left(1\right)}(u)(H_{n-1}^{\left(1\right)}(u)-H_{n+1}^{\left(1\right)}(u))}{u^{2}}\;du\\ & \;+\int_{v}^{\infty }\frac{H_{n}^{\left(1\right)}(u)(H_{n-1}^{\left(2\right)}(u)-H_{n+1}^{\left(2\right)}(u))}{u^{2}}\;du)dv, \end{array}$$

which completes the rewriting of all the integrals in *α*–*δ* to make them easy to evaluate.

## Appendix C. Convergence of the method described in §4

The asymptotic expansion (4.2) includes terms up to *O*(*ϵ*^2^), and so one might expect the error in determining each scattering coefficient to be of the order of the discarded terms, i.e. *O*(*ϵ*^4^). However, the asymptotic expansion is not the only source of error in the method. With reference to equation (4.19), we note that *a*_*n*_ can be represented schematically as

C 1 $$a_{n}=(A_{n}^{\left(0\right)}+\epsilon^{2}A_{n}^{\left(2\right)})f_{n}(b)+(B_{n}^{\left(0\right)}+\epsilon^{2}B_{n}^{\left(2\right)})f_{n}^{\prime }(b)+O(\epsilon^{4}),$$

where $A_{n}^{\left(0\right)}$, $A_{n}^{\left(2\right)}$, $B_{n}^{\left(0\right)}$ and $B_{n}^{\left(2\right)}$ can be derived by series expanding *δ*/Δ and −*β*/Δ.

Because *f*_*n*_(*b*) is derived computationally, its value will include some level of numerical error $e_{n}^{\left(1\right)}(b)$, say, so that we can write

C 2 $$f_{n}(b)={\hat{f}}_{n}(b)+e_{n}^{\left(1\right)}(b),$$

where ${\hat{f}}_{n}(b)$ is the exact value of *f*_*n*_(*b*). The function *f*_*n*_(*b*) is derived using a shooting method, so the error $e_{n}^{\left(1\right)}(b)$ will remain small until, at some very large value of *b*, it will start to grow. Upon substituting (C 2) into (C 1), we obtain

C 3 $$a_{n}=(A_{n}^{\left(0\right)}+\epsilon^{2}A_{n}^{\left(2\right)})({\hat{f}}_{n}(b)+e_{n}^{\left(1\right)}(b))+(B_{n}^{\left(0\right)}+\epsilon^{2}B_{n}^{\left(2\right)})({\hat{f}}_{n}^{\prime }(b)+e_{n}^{\left(2\right)}(b))+O(\epsilon^{4}),$$

where $e_{n}^{\left(2\right)}(b)$ is the numerical error in determining the value of $f_{n}^{\prime }(b)$, which we assume to be of the same size as $e_{n}^{\left(1\right)}(b)$.

Now let us series expand the true value of the *n*th-order scattering coefficient ${\hat{a}}_{n}$:

C 4 $${\hat{a}}_{n}=({\hat{A}}_{n}^{\left(0\right)}+\epsilon^{2}{\hat{A}}_{n}^{\left(2\right)}){\hat{f}}_{n}(b)+({\hat{B}}_{n}^{\left(0\right)}+\epsilon^{2}{\hat{B}}_{n}^{\left(2\right)}){\hat{f}}_{n}^{\prime }(b)+O(\epsilon^{4}),$$

where ${\hat{A}}_{n}^{\left(0\right)}$, ${\hat{A}}_{n}^{\left(2\right)}$, ${\hat{B}}_{n}^{\left(0\right)}$ and ${\hat{B}}_{n}^{\left(2\right)}$ are the true values of $A_{n}^{\left(0\right)}$, $A_{n}^{\left(2\right)}$, $B_{n}^{\left(0\right)}$ and $B_{n}^{\left(2\right)}$, respectively. The terms $A_{n}^{\left(0\right)}$ and $B_{n}^{\left(0\right)}$ in (C 3) should be exact as their evaluation does not require the numerical solution of any integrals. The terms $A_{n}^{\left(2\right)}$ and $B_{n}^{\left(2\right)}$ will, however, involve some level of numerical error ($d_{n}^{\left(1\right)}$ and $d_{n}^{\left(2\right)}$, say, which we assume are of the same order of magnitude as each other), and since the evaluation of the relevant integrals does not depend on a shooting method, we do not expect these to change with increasing *b*. Hence, for our purposes we have

C 5 $$A_{n}^{\left(0\right)}={\hat{A}}_{n}^{\left(0\right)},\;A_{n}^{\left(2\right)}={\hat{A}}_{n}^{\left(2\right)}+d_{n}^{\left(1\right)},\;B_{n}^{\left(0\right)}={\hat{B}}_{n}^{\left(0\right)}\;\mathrm{and}\;B_{n}^{\left(2\right)}={\hat{B}}_{n}^{\left(2\right)}+d_{n}^{\left(2\right)}.$$

Therefore, if we subtract (C 3) from equation (C 4), we obtain the total error that arises from the method described in §4:

C 6 $$\begin{array}{rl} E_{n}=|a_{n}^{\mathrm{exact}}-a_{n}| & =|A_{n}^{\left(0\right)}e_{n}^{\left(1\right)}(b)+\epsilon^{2}e_{n}^{\left(1\right)}(b)A_{n}^{\left(2\right)}+\epsilon^{2}d_{n}^{\left(1\right)}{\hat{f}}_{n}(b)+\epsilon^{2}e_{n}^{\left(1\right)}(b)d_{n}^{\left(1\right)}\\ & \;+B_{n}^{\left(0\right)}{\hat{e}}_{n}^{\left(2\right)}(b)+\epsilon^{2}{\hat{e}}_{n}^{\left(2\right)}(b)B_{n}^{\left(2\right)}+\epsilon^{2}d_{n}^{\left(2\right)}{\hat{f}}_{n}^{\prime }(b)+\epsilon^{2}e_{n}^{\left(2\right)}(b)d_{n}^{\left(2\right)}+O(\epsilon^{4})|. \end{array}$$

For small values of *b*, the $O(e_{n}^{\left(j\right)}(b))$ and $O(\epsilon^{2}d_{n}^{\left(j\right)})$, *j*=1,2, terms in equation (C 6) will be negligible and so the *O*(*ϵ*^4^) error terms will dominate. Thus, the method will converge like 1/*b*^4^; however, as *b* increases the *O*(*ϵ*^4^) terms will rapidly decrease in size until they are smaller than the $O(\epsilon^{2}d_{n}^{\left(j\right)})$ terms. Therefore, the solution will then converge at a rate proportional to 1/*b*^2^. For larger values of *b*, these terms will also decay to the point where they are smaller than $O(e_{n}^{\left(j\right)}(b))$ quantities. The latter will offer a very small constant error to the solution until, at a very large value of *b*, the shooting method will fail and the error will start to grow.

The key to selecting *b* is to choose a value that is large enough so that the *O*(*ϵ*^4^) and $O(\epsilon^{2}d_{n}^{\left(j\right)})$ errors are insignificant, but small enough that the $O(e_{n}^{\left(j\right)}(b))$ error is also insignificant. The magnitude of the $O(e_{n}^{\left(j\right)}(b))$ terms can be decreased (and therefore the error of the overall scheme decreased) by increasing the accuracy of the numerical solver; however, this will, of course, increase computation times.

## Appendix D. Scattering cross section

The scattered power flow through a surface *S* surrounding the cavity may be written [39] as

D 1 $$P_{\mathrm{out}}=\int_{S}{\text{P}}_{\mathrm{out}}\cdot \text{dA},$$

where **dA**=**n** d*A* is an incremental area element, **n** is an outer unit normal to the surface *S* and

D 2 $${\text{P}}_{\mathrm{out}}=-\frac{\partial {\text{u}}_{\mathrm{out}}}{\partial t}\cdot \tau_{\mathrm{out}}$$

is a vector that is analogous to the Poynting vector for electromagnetic waves, where **u**_out_ represents the scattered part of **u**, and ***τ***_out_ is the part of the incremental Cauchy stress (defined in (A 11)) associated with the scattered waves. We shall choose *S* to be a cylinder of unit axial length that is concentric with the cavity and with radius *r**≫*a*, so that

D 3 $$\text{n}={\text{e}}_{r}\;\mathrm{and}\;dA=r^{*}d\theta \;dz.$$

Substituting (D 2) and (D 3) into (D 1), we obtain

D 4 $$P_{\mathrm{out}}=\int_{0}^{1}\int_{0}^{2\pi }\left(-\frac{\partial w_{\mathrm{out}}}{\partial t}\tau_{zr}^{\mathrm{out}}r^{*}\right)d\theta dz=\int_{0}^{2\pi }\left(-\frac{\partial w_{\mathrm{out}}}{\partial t}\tau_{zr}^{\mathrm{out}}r^{*}\right)d\theta ,$$

where *w*_out_ is defined via **u**_out_=*w*_out_**e**_*z*_, and $\tau_{zr}^{\mathrm{out}}$ is the (*z*,*r*)th component of ***τ***_out_. Because, in general, we do not have explicit expressions for **u** and ***τ***, we shall use their far-field forms, so that

D 5 $$w_{\mathrm{out}}∼\text{Re}\left(\sum_{n=0}^{\infty }\epsilon_{n}i^{n}a_{n}H_{n}^{\left(1\right)}(kr^{*})\cos(n\theta )\;e^{-i\omega t}\right)\;\mathrm{and}\;\tau_{\mathrm{out}}=\mu_{r}^{*}\frac{\partial w_{\mathrm{out}}}{\partial r},$$

where

D 6 $$\mu_{r}^{*}=\lim_{r\to \infty }\mu_{r}(r).$$

The time-averaged scattered power is defined as follows:

D 7 $$\begin{array}{rl} {\bar{P}}_{\mathrm{out}} & =\frac{\omega }{2\pi }\int_{0}^{2\pi /\omega }P_{\mathrm{out}}\;dt \end{array}$$

D 8 $$\begin{array}{rl} & =\frac{\omega }{2\pi }\int_{0}^{2\pi /\omega }\int_{0}^{2\pi }\left(-\frac{\partial w_{\mathrm{out}}}{\partial t}\tau_{zr}^{\mathrm{out}}r^{*}\right)d\theta \;dt \end{array}$$

D 9 $$\begin{array}{rl} & =-\frac{\omega \mu_{r}^{*}r^{*}}{2\pi }\int_{0}^{2\pi /\omega }\int_{0}^{2\pi }\left(\frac{\partial w_{\mathrm{out}}}{\partial t}\frac{\partial w_{\mathrm{out}}}{\partial r}\right)d\theta \;dt. \end{array}$$

Upon substituting (D 5) into (D 6), and letting $r^{*}\to \infty$, it can be shown that, after substantial algebraic simplification,

D 10 $${\bar{P}}_{\mathrm{out}}=2\mu_{r}^{*}\omega \sum_{n=0}^{\infty }\epsilon_{n}|a_{n}{|}^{2}.$$

The intensity of the incoming wave is defined as the time-averaged power passing through a unit square, which using the same methods as above can be obtained as follows:

D 11 $$I={\bar{P}}_{\mathrm{in}}=\frac{\mu_{r}^{*}k\omega }{2}.$$

The scattering cross section, then, is defined as follows:

D 12 $$Q=\frac{{\bar{P}}_{\mathrm{out}}}{I}=\frac{4}{k}\sum_{n=0}^{\infty }\epsilon_{n}|a_{n}{|}^{2}.$$

As in Lewis *et al.* [40], however, we prefer the non-dimensionalized form of the scattering cross section:

D 13 $$q=\frac{Q}{2A}=\frac{2}{kA}\sum_{n=0}^{\infty }\epsilon_{n}|a_{n}{|}^{2}.$$

Note that we have non-dimensionalized on the *undeformed* cavity radius *A*.
